# Minimum Distribution Support Vector Clustering

**DOI:** 10.3390/e23111473

**Published:** 2021-11-08

**Authors:** Yan Wang, Jiali Chen, Xuping Xie, Sen Yang, Wei Pang, Lan Huang, Shuangquan Zhang, Shishun Zhao

**Affiliations:** 1 Key Laboratory of Symbol Computation and Knowledge Engineering, Ministry of Education, Colleague of Computer Science and Technology, Jilin University, Changchun 130012, China; wy6868@jlu.edu.cn (Y.W.); jiali19@mails.jlu.edu.cn (J.C.); xiexp21@mails.jlu.edu.cn (X.X.); ystop2020@gmail.com (S.Y.); shuangquan18@mails.jlu.edu.cn (S.Z.); 2 School of Artificial Intelligence, Jilin University, Changchun 130012, China; 3 School of Mathematical and Computer Sciences, Heriot-Watt University, Edinburgh EH14 4AS, UK; w.pang@hw.ac.uk; 4 College of Mathematics, Jilin University, Changchun 130012, China; zhaoss@jlu.edu.cn

**Keywords:** support vector clustering, margin theory, mean, variance, dual coordinate descent

## Abstract

Support vector clustering (SVC) is a boundary-based algorithm, which has several advantages over other clustering methods, including identifying clusters of arbitrary shapes and numbers. Leveraged by the high generalization ability of the large margin distribution machine (LDM) and the optimal margin distribution clustering (ODMC), we propose a new clustering method: minimum distribution for support vector clustering (MDSVC), for improving the robustness of boundary point recognition, which characterizes the optimal hypersphere by the first-order and second-order statistics and tries to minimize the mean and variance simultaneously. In addition, we further prove, theoretically, that our algorithm can obtain better generalization performance. Some instructive insights for adjusting the number of support vector points are gained. For the optimization problem of MDSVC, we propose a double coordinate descent algorithm for small and medium samples. The experimental results on both artificial and real datasets indicate that our MDSVC has a significant improvement in generalization performance compared to SVC.

## 1. Introduction

Cluster analysis groups a dataset into clusters according to the correlations of data. To date, many clustering algorithms have emerged, such as plane-based clustering algorithm, spectral clustering, density-based DBSCAN [1], OPTICS [2], Density Peak algorithm (DP) characterizing the center of clusters [3], and partition-based k-means algorithm [4]. In particular, the support vector machine (SVM) has become an important tool for data mining. As a classical machine learning algorithm, SVM can well address the issue of local extremum and high dimensionality of data in the process of model optimization, and it makes data separable in feature space through nonlinear transformation [5].

In particular, Tax and Duin proposed a novel method in which the decision boundaries are constructed by a set of support vectors, the so-called support vector domain description (SVDD) [6]. Leveraged by the kernel theory and SVDD, support vector clustering (SVC) was proposed based on contour clustering, which has many advantages over other clustering algorithms [7]. SVC is robust to noise and does not need to pre-specify the number of clusters in advance. For SVC, it is feasible to adjust its parameter C to obtain better performance, but this comes at the cost of increasing outliers, and it only introduces a soft boundary for optimization. Several insights into understanding the features of SVC have been offered in [8,9]. After studying the relevant literature, we found that these insights mainly cover two aspects: the first aspect is the selection of parameters *q* and *C*. Lee and Daniels chose a method similar to a secant to generate monotone increasing sequences of *q* and establish the monotone function of *q* and radius R, which can be applied to high dimensions; the second aspect is optimizing the cluster assignments. Considering the high cost of the second stage of SVC, several methods have been proposed for improving the cluster partition of SVC. First, Ben et al. improved the original Complete Graph (CG) partition by using the adjacency matrix partition based on SV points, which simplified the original calculation, but this method failed to avoid random sampling. Yang et al. elaborated on the Proximity Graph (PG) to model the proximity structure of the m samples with time complexity of O(m) or O(mlog(m)). However, the complexity of this algorithm increases with the increase in dimensionality [10]. Lee et al. studied a cone cluster labeling (CCL) method by using the geometry of the feature space to assign clusters in the data space. If two cones intersect, the samples in these cones belong to the same cluster [9]. However, the performance of CCL is sensitive to kernel parameter q for the cones decided by q. More recently, Peng et al. designed a partition method that utilized the clustering algorithm of similarity segmentation-based point sorting (CASS-PS) and considered the geometrical properties of support vectors in the feature space to avoid the downsides of SVC and CASS-PS [11]. However, CASS-PS is sensitive to the number and distribution of the support vector points recognized. Jennath and Asharaf proposed an efficient cluster assignment algorithm for SVC using the similarity of feature set for data points utilizing an efficient MEB approximation algorithm [12].

It is well known from the margin theory that maximizing the minimum margin is often not the best way for further improving the learning performance. Regarding this, the introduction of the margin mean and margin variance in distribution can make the model achieve better generalization performance, as revealed by Gao and Zhou [13,14]. In classification and regression analysis, there are many methods for improving the learning performance by considering the statistical information of the data. Zhang and Zhou proposed the large margin distribution machine (LDM) and optimal margin distribution machine (ODM) for data classification, which adjusted the mean and variance to improve the performance of the model [15,16]. In regression analysis, MDR, ε-SVR, LDMR, and v-MDAR considers the marginal distribution to achieve better performance. MDR, proposed by Liu et al., minimizes the regression deviation mean and the regression deviation variance, which introduced the statistics of regression deviation into ε-SVR [17]. To deal with this issue, Wang et al. characterized the absolute regression deviation mean and the absolute regression deviation variance and proposed the v-minimum absolute deviation distribution regression (v-MADR) machine [18]. However, it is not very appropriate when both positive-label and negative-label samples are present. Inspired by LDM, Rastogi et al. also proposed a large margin distribution machine-based regression model (LDMR) [19].

In clustering analysis, for a good clustering, when the labels are consistent with the clustering results, SVM can obtain a larger minimum margin. Inspired by this, maximum margin clustering (MMC) considered the large margin heuristic from SVM and added the maximum margin to all possible markers [20]. Improved versions of MMC are also proposed [21]. The optimal margin distribution clustering (ODMC) proposed by Zhang et al. forms the optimal marginal distribution during the clustering process, which characterizes the margin distribution by the first- and second-order statistics. It also has the same convergence rate as state-of-the-art cutting plane-based algorithms [22].

The success of the aforementioned models suggests that there may still exist room for further improving SVC. These models do not involve the improvement in the generalization performance of SVC, that is, the reconstruction of hyperplane, when the distribution of data is fixed in feature space. In this research, we propose a novel approach called minimum distribution support vector clustering (MDSVC), and our novel contributions are as follows:

- We characterize the envelope radius of minimum hypersphere by the first- and second-order statistics, i.e., the mean and variance. By minimizing these two statistics, it can avoid the problem of too many or too few support vector points caused by the inappropriate kernel width coefficient q to some extent, form a better cluster contour, and, thus, improve the accuracy.
- We enhance the generalization ability and robustness of the algorithm by introducing these statistics while the distribution of data is fixed for the given *q* in feature space.
- We further prove that our method has better performance inspired by the expectation of the probability of test error proposed in SVDD.
- We customize a dual coordinate descent (DCD) algorithm to optimize the objective function of MDSVC for our experiments.

The remainder of this paper is organized as follows. Section 2 introduces the notations, the recent progress in the margin theory, and the SVC algorithm. In Section 3, we present the MDSVC algorithm, which minimizes the mean and the variance, and propose a DCD algorithm to solve the objective function of MDSVC. Section 4 reports our experimental results on both artificial and real datasets. We discuss our method in Section 5 and draw conclusions in Section 6.

## 2. Background

Suppose $D=\left[x_{1},\ldots ,x_{m}\right]$ is a dataset of m samples, and each column is a sample of a d-dimensional vector. *ϕ*(***x***) is the mapping function induced by a kernel *k*, i.e., $k\left(x_{i},x_{j}\right)=\phi {\left(x_{i}\right)}^{T}\phi \left(x_{j}\right)$. SVC used the nonlinear Gaussian kernel function $k\left(x_{i},x_{j}\right)=\exp\left(-q*\|x_{i}-x_{j}\|{}^{2}\right)$. Obviously, we have k(x,x)=1. Both MDSVC and SVC aim to obtain the radius R of the sphere, center a of the hypersphere, and the radius of each point in feature space. Formally, we denote *X* the matrix whose *i*-th column is *ϕ*(***x_i_***), i.e., $x=\left[\phi \left(x_{1}\right),\ldots ,\phi \left(x_{m}\right)\right]$. In this paper, we use the Gaussian kernel as our nonlinear transformation approach to map data points to feature space.

### Recent Progress in Margin Theory

Recent margin theory indicates that maximizing the minimum margin may not lead to an optimal result and better generalization performance. In the SVC algorithm, when the kernel width coefficient q is selected, the distribution of data points mapped to the feature space is determined. If the distribution of boundary data is different from that of internal data, the hyperplane constructed by SVC may not make better use of the data information, thus reducing the performance of SVC. Additionally, we note that SVC is always overfitting with too many support vector points in practice. Gao and Zhou have already demonstrated that marginal distribution is critical to the generalization performance [13]. The high generalization ability of margin has been shown in v-MADR, which minimizes both the absolute regression deviation mean and the absolute regression deviation variance [18]. We also note that SVC can be regarded as a binary classifier divided by the division hyperplane. Inspired by the aforementioned research, we introduce the mean and variance of the marginal distribution and minimize them to reduce the number of support vector points.

For the convenience of readers, a more detailed description of SVC is presented in Appendix A.

## 3. Minimum Distribution Support Vector Clustering

In this section, we briefly delineate the process of MDSVC, including three subsections, the formula of MDSVC, which minimizes both the mean and the variance, the optimization algorithms based on dual coordinate descent method, and the statistical property of MDSVC that shows the upper bound of the expectation of error. In this research, as mentioned before, we take the Gaussian kernel as a nonlinear transformation approach to map data points to the feature space, and then we derive *k*(***x***, ***x***) = 1, which is critical for us to simplify the variance and solve the objective function. In addition, we define the mean and variance based on the Euclidean distance. The reason we employ the Euclidean distance is that we can take the objective function as the convex quadratic programming function and the Euclidean norm represents the actual distance between two points rather than the distance on the surface.

**Figure 1:**
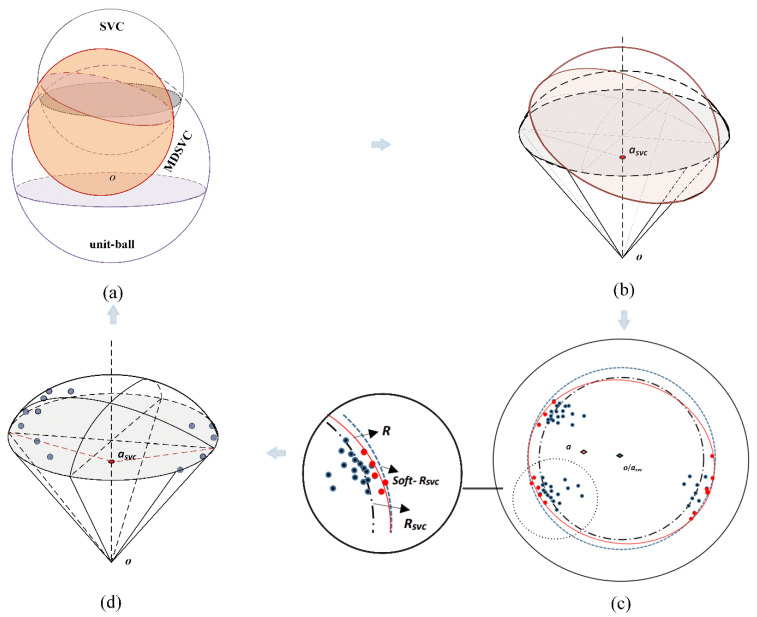
(**a**) Hyperplanes of SVC and MDSVC. (**b**) Two caps formed by SVC and MDSVC with the unit-ball respect tively. (**c**) Top view of Figure 1a. (**d**) Data distribution in the cap.

We delineate the idea of our algorithm in the feature space in Figure 1 roughly, and more detailed descriptions are given in Section 3.1.1 and Section 3.1.2. First, the hyperplanes of MDSVC, SVC, and the unit ball are shown in Figure 1a. By characterizing and minimizing our mean and variance, we can, thus, have the hypersphere of MDSVC as an inclined curved surface in the feature space, as indicated in red in Figure 1a. The intersection of the SVC’s hypersphere and the unit sphere is a cap-like area. We further illustrate the main difference between MDSVC and SVC through a lateral view and top view, which are shown in Figure 1b,c, respectively. Figure 1b is the schematic diagram of the MDSVC’s Cap and the SVC’s Cap. We can find that the center ***a*** of MDSVC’s hypersphere moves away from the center of the ball and inclines to the distribution of the overall data because of the mean and variance. In Figure 1c, we use *Soft-R_svc_* to represent the soft boundary of SVC. The centers of the three spheres, namely the unit ball, SVC’s hypersphere, and MDSVC’s hypersphere, are denoted by ***o***, ***a_svc_***, and ***a***, respectively. We also use red points to indicate the SVs of MDSVC. As shown in Figure 1c, we can see how the boundary of MDSVC *R* is determined. Finally, we use Figure 1d to show the distribution of data points and the details of the Cap formed by SVC.

### 3.1. Formula of MDSVC

#### 3.1.1. Preliminary

Let *ϕ*(***x***) be the mapping function induced by a kernel *k*, i.e., $k\left(x_{i},x_{j}\right)=\phi {\left(x_{i}\right)}^{T}\phi \left(x_{j}\right)$. In the feature space, we use the Gaussian kernel, and we derive *k*(***x***, ***x***) = 1. The distance between ***a*** and ***x*** is $\|\phi \left(x\right)-a{\|}^{2}$, where ‖.‖ is the Euclidean norm and ***a*** is the center of the sphere. We denote ***X*** as the matrix whose *i*-th column is *ϕ*(***x_i_***). In what follows in the rest of this subsection, we first give the definitions of statistics of mean and variance in clustering; we then present Theorems 1 and 2 to facilitate the formation of the variance; next, we employ the mean and variance (Equations (1) and (2)) to obtain and elucidate the final formula as a convex quadratic programming problem.

**Definition** **1.**  *The margin mean is defined as follows.*

(1) $$\overset{-}{\gamma }=\frac{1}{m}\sum_{i}{\left\|\phi (x_{i})-a\right\|}^{2}=1-\frac{2}{m}a^{T}Xe+a^{2}$$

where ***e*** stands for the all-one column vector of *m* dimensions. Because we use the Gaussian kernel, we have ***k***(***x***, ***x***) = 1, which can facilitate the calculation. The reason for choosing this form of mean is that we incline to make the center of the MDSVC’s sphere close to the denser part of the samples. Next, we define the margin variance.

**Definition** **2.**  *The margin variance is defined as follows.*

(2) $$\begin{array}{l} \overset{\wedge }{\gamma }=\frac{1}{m^{2}}\sum_{i=1}^{m}\sum_{j=1}^{m}({\left\|\phi (x_{i})-a\right\|}^{2}-{\left\|\phi (x_{j})-a\right\|}^{2}{)}^{2}\\ \;\;=\frac{4}{m^{2}}\sum_{i=1}^{m}\sum_{j=1}^{m}(a^{T}\phi (x_{i})-a^{T}\phi (x_{j}){)}^{2}\\ \;\;=\frac{4}{m^{2}}\sum_{i=1}^{m}\sum_{j=1}^{m}(a^{T}\phi (x_{i})\phi {\left(x_{i}\right)}^{T}a-2a^{T}\phi (x_{i})\phi {\left(x_{j}\right)}^{T}a\;+\;a^{T}\phi (x_{j})\phi {\left(x_{j}\right)}^{T}a)\\ \;\;=\frac{8}{m}\sum_{i=1}^{m}a^{T}\phi (x_{i})\phi {\left(x_{i}\right)}^{T}a\;+\;\frac{8}{m^{2}}\sum_{i=1}^{m}\sum_{j=1}^{m}a^{T}\phi (x_{i})\phi {\left(x_{j}\right)}^{T}a \end{array}$$

The variance considers the distribution of the overall data rather than the distribution of SVs. Note that if we only characterize the mean in our method, the hyperplane would incline to dense clusters and there may appear more support vectors for the high density of the clusters, which will result in unbalance. However, we should realize that the mean is just the first step to adjusting the sphere of MDSVC. Next, we introduce the variance to adjust the boundary with less volatility. We can find that the variance quantifies the scatter of clustering. Additionally, we denote kernel matrix *Q = X^T^X*, where $Q_{ij}=k\left(x_{i},x_{j}\right)=\phi {\left(x_{i}\right)}^{T}\phi \left(x_{j}\right)$. Note that $\phi \left(x_{i}\right)\phi {\left(x_{j}\right)}^{T}$, different from $\phi {\left(x_{i}\right)}^{T}\phi \left(x_{j}\right)$, is difficult to obtain due to its complicated form, so we have to use an alternative way to address this issue. Thus, we use the following Theorem 1. Note that the formula of variance can be further simplified, so we employ Theorem 2 to elucidate and facilitate the form of the variance. Finally, we obtain the simplified form for the margin variance as in Equation (8).

**Theorem** **1.**  *The center of hypersphere a can be represented as follows,*

(3) $$a=\;\sum_{i=1}^{m}\alpha_{i}\phi (x_{i})=X\alpha$$

**Proof** **of** **Theorem** **1.**  Suppose that ***a*** can be decomposed into the span of *ϕ*(***x_i_***) and an orthogonal vector ***v***, that is

(4) $$a=\;\sum_{i=1}^{m}\alpha_{i}\phi (x_{i})\;+\;v=X\alpha \;+v,\;\;\;\;\;\;\;\;\;\;\;\alpha ={\left[\alpha_{1},\ldots ,\alpha_{m}\right]}^{T}$$

where ***v*** satisfies $\phi {\left(x_{i}\right)}^{T}v=0$ for all *i*, i.e., $x^{T}v=0$. Then we have the following formula

(5) $$a^{2}=\alpha^{T}X^{T}X\alpha +v^{T}v\geq \alpha^{T}X^{T}X\alpha$$

Therefore, when minimizing ***a***, ***v*** = 0 does not affect its value. The formula of mean is then derived as follows

$$\begin{array}{l} \overset{-}{\gamma }=\frac{1}{m}\sum_{i}{\left\|\phi (x_{i})-a\right\|}^{2}=1-\frac{2}{m}\alpha^{T}X^{T}Xe+\alpha^{T}X^{T}X\alpha +v^{T}v\\ \;\;\geq 1-\frac{2}{m}\alpha^{T}X^{T}Xe+\alpha^{T}X^{T}X\alpha \end{array}$$

From the aforementioned formula, the mean is equivalent to modulus ***a*** in optimization, that is, $\overset{-}{\gamma }\Leftrightarrow a^{T}a$. For variance, we have the following form

(6) $$\begin{array}{l} \overset{\wedge }{\gamma }=\frac{1}{m^{2}}\sum_{i=1}^{m}\sum_{j=1}^{m}({\left\|\phi (x_{i})-a\right\|}^{2}-{\left\|\phi (x_{j})-a\right\|}^{2}{)}^{2}\\ \;\;=\frac{8}{m}\sum_{i=1}^{m}a^{T}\phi (x_{i})\phi {\left(x_{i}\right)}^{T}a\;+\;\frac{8}{m^{2}}\sum_{i=1}^{m}\sum_{j=1}^{m}a^{T}\phi (x_{i})\phi {\left(x_{j}\right)}^{T}a\\ \;\;=\frac{8}{m}\sum_{i=1}^{m}\alpha^{T}X^{T}\phi (x_{i})\phi {\left(x_{i}\right)}^{T}X\alpha +\frac{8}{m^{2}}\sum_{i=1}^{m}\sum_{j=1}^{m}\alpha^{T}X^{T}\phi (x_{i})\phi {\left(x_{j}\right)}^{T}X\alpha \end{array}$$

Thus, the variance is independent of ***v***. The rest of the optimization objectives are also independent of ***v***. Based on all of the aforementioned equations, ***a*** can be represented as the form of Equation (3). □

**Theorem** **2.**  $Q_{i}Q_{i}{}^{T}$, $\overset{m}{\underset{i=1}{\sum }}\overset{m}{\underset{j=1}{\sum }}Q_{i}Q_{j}{}^{T}$*, **H**, **P**, **QG** are symmetric matrices where*

$$Q_{i}=\left[\begin{array}{c} k(x_{1},x_{i})\\ \vdots \\ k(x_{m},x_{i}) \end{array}\right],H=\frac{8\lambda_{2}}{m}\sum_{i=1}^{m}Q_{i}Q_{i}{}^{T}\;P=\frac{8\lambda_{2}}{m^{2}}\sum_{i=1}^{m}\sum_{j=1}^{m}Q_{i}Q_{j}{}^{T},G={\left((\lambda_{1}+1)Q+H+P\right)}^{-1}Q\;{\left((\lambda_{1}+1)Q+H+P\right)}^{-1}\;\mathrm{refers}\;\mathrm{to}\;\mathrm{the}\;\mathrm{inverse}\;\mathrm{matrix}\;\mathrm{of}\;(\lambda_{1}+1)Q+H+P$$

**Proof** **of** **Theorem** **2.**  $Q_{i\left(m\times 1\right)}$ is a column vector of the kernel matrix ***Q*** with the following form

$$Q_{i(m\times 1)}=\left[\begin{array}{c} k(x_{1},x_{i})\\ \vdots \\ k(x_{m},x_{i}) \end{array}\right]\;Q_{i}Q_{i}{}^{T}=\left[\begin{array}{c} k(x_{1},x_{i})\\ \vdots \\ k(x_{m},x_{i}) \end{array}\right]\left[\begin{array}{ccc} k(x_{1},x_{i}) & \cdots & k(x_{m},x_{i}) \end{array}\right]\;\;\;\;\;\;\;=\left[\begin{array}{ccc} k{\left(x_{1},x_{i}\right)}^{2} & \cdots & k(x_{1},x_{i})k(x_{m},x_{i})\\ \vdots & \ddots & \vdots \\ k(x_{1},x_{i})k(x_{m},x_{i}) & \cdots & k{\left(x_{m},x_{i}\right)}^{2} \end{array}\right]$$

Note that $Q_{i}Q_{i}{}^{T}$ is a symmetric matrix from the above form. Obviously, $\overset{m}{\underset{i=1}{\sum }}\overset{m}{\underset{j=1}{\sum }}Q_{i}Q_{j}{}^{T}\;$ is a symmetric matrix. Therefore, H and P are both symmetric matrices. We deduce ***QG*** as follows

$$\begin{array}{l} QG=Q{\left((\lambda_{1}+1)Q+H+P\right)}^{-1}Q\\ \Rightarrow {\left(QG\right)}^{T}={\left(Q((\lambda_{1}+1)Q+H+P\right)}^{-1}Q{)}^{T}\\ \;\;\;\;\;\;\;\;\;\;=Q{\left((\lambda_{1}+1)Q+H+P)T\right)}^{-1}Q=Q{\left((\lambda_{1}+1)Q+H+P\right)}^{-1}Q\\ \Rightarrow {\left(QG\right)}^{T}=G^{T}Q=QG \end{array}$$

Therefore, ***QG*** is a symmetric matrix. □

According to Theorem 1, we have the following form of mean and variance

(7) $$\overset{-}{\gamma }=\frac{1}{m}\sum_{i}{\left\|\phi (x_{i})-a\right\|}^{2}=1-\frac{2}{m}\alpha^{T}Qe+\alpha^{T}Q\alpha$$

(8) $$\begin{array}{l} \overset{\wedge }{\gamma }=\frac{8}{m}\alpha^{T}\sum_{i=1}^{m}Q_{i}Q_{i}{}^{T}\alpha +\frac{8}{m^{2}}\alpha^{T}\sum_{i=1}^{m}\sum_{j=1}^{m}Q_{i}Q_{j}{}^{T}\alpha \\ \;\;=\alpha^{T}(\frac{8}{m}\sum_{i=1}^{m}Q_{i}Q_{i}{}^{T}+\frac{8}{m^{2}}\sum_{i=1}^{m}\sum_{j=1}^{m}Q_{i}Q_{j}{}^{T})\alpha \end{array}$$

#### 3.1.2. Minimizing the Mean and Variance

Referring to the above subsections, we define the formula of MDSVC as follows

(9) $$\begin{array}{l} \min_{R,a}R^{2}+\lambda_{1}\overset{-}{\gamma }+\lambda_{2}\overset{\wedge }{\gamma }+C\sum_{i=1}^{m}\xi_{i}\\ s.t.{\left\|\phi (x_{i})-a\right\|}^{2}\leq R^{2}+\xi_{i},\\ \;\;\;\xi_{i}\geq 0 \end{array}$$

Consider that the center ***a*** of the sphere is closer to the denser part in the feature space as minimizing the mean, and then we minimize the value of *λ*_2_ to make more points closer to ***a***, resulting in fewer support vector points. Next, we simplify Equation (9).

Based on Theorem 1, Equation (9) leads to

(10) $$\begin{array}{l} \min_{R,\alpha }R^{2}+\alpha^{T}(\lambda_{1}Q+H+P)\alpha -\frac{2\lambda_{1}}{m}e^{T}Q\alpha +C\sum_{i=1}^{m}\xi_{i}\\ s.t.{\left\|\phi (x_{i})-X\alpha \right\|}^{2}\leq R^{2}+\xi_{i},\\ \;\;\;\xi_{i}\geq 0 \end{array}$$

By introducing Lagrange multipliers *β_i_*, *μ_i_*, the Lagrange function of Equation (12) is given as follows

(11) $$\begin{array}{l} L(R,\alpha ,\xi ,\beta ,\mu )=\alpha^{T}{\left((\lambda +1\right)}_{1}Q+H+P)\alpha \\ \;\;\;\;\;\;\;-(\frac{2\lambda_{1}}{m}e^{T}Q+2\beta^{T}Q)\alpha +R^{2}(1-\sum_{i=1}^{m}\beta_{i})+\sum_{i=1}^{m}(C-\mu_{i}-\beta_{i})\xi_{i}\; \end{array}$$

By setting the partial derivatives {R,α,ξ} to zero for satisfying the KKT conditions, we have the following equations of derivatives

(12) $$\frac{\partial L}{\partial R}=2R-2R\sum_{i=1}^{m}\beta_{i}=0$$

(13) $$\frac{\partial L}{\partial \alpha }=2\alpha^{T}((\lambda_{1}+1)Q+H+P)-(\frac{2\lambda_{1}}{m}e^{T}Q+2\beta^{T}Q)=0$$

(14) $$\frac{\partial L}{\partial \xi_{i}}=C-\mu_{i}-\beta_{i}=0\;$$

Thus, we adopt $G={\left((\lambda_{1}+1)Q+H+P\right)}^{-1}Q$, where ${\left((\lambda_{1}+1)Q+H+P\right)}^{-1}$ refers to the inverse matrix of $\left((\lambda_{1}+1)Q+H+P\right)$. On the basis of these equations, we obtain vector ***A*** as follows

(15) $$A=\frac{\lambda_{1}}{m}{\left((\lambda_{1}+1)Q+H+P\right)}^{-1}Qe=\frac{\lambda_{1}}{m}Ge\;$$

Substituting Equation (15) into Equation (13), we thus have

(16) α=A+Gβ 

By substituting Equations (12)–(14) into Equation (11), Equation (11) is re-written as follows

(17) $$\begin{array}{l} L(\beta )={\left(A+G\beta \right)}^{T}((\lambda_{1}+1)Q+H+P)(A+G\beta )-(\frac{2\lambda_{1}}{m}e^{T}Q+2\beta^{T}Q)(A+G\beta )\\ \;\;\;\;\;\;=\underset{\beta }{\min}\frac{1}{2}\beta^{T}D\beta +F\beta \end{array}$$

We notice that $G={\left((\lambda_{1}+1)Q+H+P\right)}^{-1}Q$, so ***D*** and ***F*** have the following form

(18) $$\begin{array}{l} D=4QG-2G^{T}Q=2G^{T}Q=2QG\\ F=\frac{2\lambda_{1}}{m}e^{T}QG \end{array}$$

Referring to the above equations, thus, we derive our formula of MDSVC as follows

(19) $$\begin{array}{l} \;\underset{\beta }{\min}\frac{1}{2}\beta^{T}D\beta +F\beta \;\\ \;\;\;\;\;\;\;s.t.\;0\leq \beta_{i}\leq C \end{array}$$

Based on Theorem 2, ***D*** is symmetric and consists of positive elements. We can then make a conclusion that Equation (19) is a convex quadratic problem resulting from the convex objective function and convex domain β∈[0,C]. Thus, we can solve the objective function with convex quadratic programming.

### 3.2. The MDSVC Algorithm

Due to the simple box constraint and the convex quadratic objective function of our optimization problem, we adopt the DCD algorithm to minimize one of the variables continuously and keep the other variables fixed to obtain the closed form solution. For our problem, we adjust the value of *β_i_* with a step size of *t* to make *f*(***β***) reach the minimum value, while keeping other $\beta_{k\neq i}$ unchanged. Our sub-problem is thus as follows

(20) $$\left\{ \begin{array}{l} \underset{\beta }{\min}f(\beta +te_{i})\\ 0\leq \beta_{i}+t\leq C \end{array}\right.$$

where $e_{i}={\left(0,\ldots ,1_{i},..,0\right)}_{m}{}^{T}$ denotes the vector with 1 in the *i*-th element and 0 is elsewhere. For function *f*, we have

(21) $$f(\beta +te_{i})=\frac{1}{2}d_{ii}t^{2}+\nabla f{\left(\beta \right)}_{i}t+f(\beta )$$

where $d_{ii}=e_{i}{}^{T}De_{i}$ is the diagonal entry of ***D***. Then we calculate the gradient by the following form

(22) $$\nabla f{\left(\beta \right)}_{i}=e_{i}{}^{T}D\beta +e_{i}{}^{T}F^{T}$$

As *f*(***β***) is independent of *t*, we can consider Equation (21) as a function of *t*. Hence, $f\left(\beta +te_{i}\right)$ can be transformed into a simple quadratic function of *t*. Thus, we get the minimum value of Equation (21) by setting the derivation of the aforementioned function with respect to t to zero. Therefore, *t* is represented as follows

(23) $$t=-\frac{\nabla f{\left(\beta \right)}_{i}}{d_{ii}}$$

We denote $\beta_{i}{}^{iter}$ as the value of *β_i_* at the *i*-th iteration, thus, the value of $\beta_{i}{}^{iter+1}$ can be obtained as

(24) $$\beta_{i}{}^{iter+1}=\beta_{i}{}^{iter}-\frac{\nabla f{\left(\beta \right)}_{i}}{d_{ii}}$$

Considering the box constraint $0\leq \beta_{i}\leq C$ of the problem, we can further obtain the final form of updating *β_i_*

(25) $$\beta_{i}\leftarrow \min\left(\max\left(\beta_{i}-\frac{\nabla f{\left(\beta \right)}_{i}}{d_{ii}},0\right),C\right)$$

According to Equations (16) and (19), we have ${\left[\nabla f\left(\beta \right)\right]}_{i}=2e_{i}{}^{T}Q\alpha$. Algorithm 1 (MDSVC) describes the procedure of MDSVC with the Gaussian kernel.
**Algorithm 1: MDSVC.** The DCD Algorithm for our method MDSVCStep 1. **Input**: Data set ***X***, parameters:$[\lambda_{1},\lambda_{2},C,q$], maxIter, *m* Step 2. **Initialization**: $\beta =\frac{\;\lambda_{1}}{m}e$$,\alpha =\frac{\;2\lambda_{1}}{m}Ge$$,\;d_{ii}=2e_{i}{}^{T}QGe_{i},\;G={\left(\left(\;\lambda_{1}+1\right)Q+H+P\right)}^{-1}Q$ Step 3. Iteration(1~maxIter): Iteration stops when the *β* converges. Step 3.1. Randomly disturb *β* and then get the random index *i* Step 3.2. Loop (*i* = 1, 2, …, *m*): update gradient and update *β*, *α* alternately.${\left[\nabla f\left(\beta \right)\right]}_{i}\leftarrow 2e_{i}{}^{T}Q\alpha$ $\beta_{i}{}^{temp}\leftarrow \beta_{i}$ $\beta_{i}\leftarrow \min\left(\max\left(\beta_{i}-\frac{\nabla f{\left(\beta \right)}_{i}}{d_{ii}},0\right),C\right)$ $\alpha \leftarrow \alpha +\left(\beta_{i}-\beta_{i}{}^{temp}\right)Ge_{i}$Step 4. Output: *α*, *β*.

Meanwhile, we give the analysis of the computational complexity of Algorithm MDSVC, where m denotes the number of the examples and n represents the number of features. We set maxIter to 1000 during our experiments, the time complexity of DCD, thus, can be cast as maxIter*m*m. Furthermore, we can infer that the time complexity of DCD in this paper is the sum of time complexity as shown in Table 1. Considering that *m* is much greater than *n*, thus, the time complexity of DCD is *O*(*m*^3^), and the space complexity of DCD is *O*(*m*^2^).

### 3.3. The Properties of MDSVC

We briefly introduce the properties of MDSVC in this subsection. Hereinafter, the points with 0 < *β_i_* < *C* will be referred to as support vectors (SVs); the points with *β_i_* = *C* will be called bounded support vectors (BSVs), which are the same as in SVC. Additionally, the SVDD [5] used cross-validation (leave-one-out) as the criterion to characterize the expectation of the probability of test error, and, then, they describe the expectation as follows

(26) $$E(P(error))=\frac{num(SV)}{m}$$

The above expectation is more suitable as a standard for adjusting the parameters in the experiments of SVDD rather than having a theoretical basis. It can only estimate the error of the first kind, i.e., the target class. By analyzing the above equation, we further infer that our algorithm can reduce the number of SVs to some extent compared with SVC. Thus, we can obtain better generalization performance compared with SVC theoretically. Inspired by SVDD and LDM, we give the expectation in a manner similar to the approach used in LDM.

**Theorem** **3.**  *The center. Let **β** represent the optimal solution of Equation (19) and* E[R(β)]*be the expectation of the probability of error, and then we obtain*

(27) $$E[R(\beta )]\leq \frac{E[d\sum_{i\in I_{1}}\frac{\beta_{i}{}^{*}}{2(1-R^{2})}+\left|I_{2}\right|]}{m},$$

*where* $I_{1}\equiv \{i|0<\alpha_{i}<C\}$, $I_{2}\equiv \{i|\alpha_{i}=C\}$, d=max{diag{D}}.

**Proof** **of** **Theorem** **3.** Suppose

$$\begin{array}{l} \beta^{*}=\underset{0\leq \beta \leq C}{\mathrm{argmin}}f(\beta ),\\ \beta^{i}\;\;=\underset{0\leq \beta \leq C,\beta^{i}=0}{\mathrm{argmin}}f(\beta ),\;i=1,\ldots ,m, \end{array}$$

and the parameters of the sphere are *R* and ***a***, respectively. As in [16], the expectation is calculated as below

(28) $$E[R(\beta )]=\frac{E[\gamma ((x_{1},y_{1}),\ldots ,(x_{m},y_{m}))]}{m},$$

where $\gamma \left(\left(x_{1},y_{1}\right),\ldots ,\left(x_{m},y_{m}\right)\right)$ is the number of errors produced during the leave-one-out procedure. Data points are divided into three categories. Note that if $\beta_{i}{}^{*}=0$, the point is interior in the data space. The cluster of the interior points is totally up to the SVs regardless of the assignment of the cluster in the second stage of the MDSVC procedure based on the analysis of SVDD. Hence, we consider two cases as follows:

(1) $0<\beta_{i}{}^{*}<C,$ the data is the support point according to the SVC and KKT conditions, we have
  (29) $$f(\beta^{i})-\underset{t}{min}f(\beta^{i}+te_{i})\leq f(\beta^{i})-f(\beta^{*})\leq f(\beta^{*}-\beta_{i}{}^{*}e_{i})-f(\beta^{*}),$$
  where *e_i_* is a vector with 1 in the *i*-th coordinate and 0 elsewhere. Incorporating Equation (16) into the aforementioned formula, we have $\langle \phi (x_{i},a)\rangle \leq \frac{\beta_{i}*d_{ii}}{2}$, where ***x****_i_* are SVs. Further, note that if ***x****_i_* is an SV, we have $\langle \phi (x_{i},a)\rangle =\|a{\|}^{2}=1-R^{2}$, which is a lemma proposed in CCL [9]. Thus, we rearrange $\langle \phi (x_{i},a)\rangle \leq \frac{\beta_{i}*d_{ii}}{2}$, and then obtain $1\leq \frac{\beta_{i}*d_{ii}}{2\left(1-R^{2}\right)}$.
(2) $\beta_{i}{}^{*}=C$, ***x****_i_* is the bounded SV (SVs) and must be misclassified in the leave-one-out procedure. Hence we have
  (30) $$\gamma ((x_{1},y_{1}),\ldots ,(x_{m},y_{m}))\leq d\sum_{i\in I_{1}}\frac{\beta_{i}{}^{*}}{2(1-R^{2})}+\left|I_{2}\right|$$
  where $I_{1}\equiv \{i|0<\alpha_{i}<C\}$, $I_{2}\equiv \{i|\alpha_{i}=C\}$, d=max{diag{D}}. Taking the mean of both sides of Equation (30) and with Equation (28), we finally obtain the result that Equation (27) holds. □

## 4. Experimental Study

In this section, MDSVC is compared with k-means (KM) [4], optimal margin distribution clustering (ODMC) [22], spectral clustering (SC) [23], mean shift (MS) [24], and hierarchical clustering (HC) [25]. We adopt the results of K-means acting as a baseline rather than maximum margin clustering (MMC) [20] since it could not return results in a reasonable time for most datasets. We experimentally evaluate the performance of our MDSVC compared with the original algorithms of SVC on classic artificial datasets and several medium-sized datasets; that is, we focus on the difference between MDSVC and SVC. Table 2 summarizes the statistics of these data sets. All real-world datasets used for our experiments can be found at UCI (http://archive.ics.uci.edu/ml, 2 February 2021). In Table 2, all of the samples of artificial datasets, namely convex, dbmoon, and ring, are added with Gaussian noises, which are representative of different types of datasets. All algorithms are implemented with MATLAB R2021a on a PC with a 2.50 GHz CPU and 64 GB memory.

### 4.1. Evaluation Criteria

To evaluate the performance of MDSVC, we use two external indicators, clustering accuracy (Acc) and Adjusted Rand Index (ARI), as our performance metrics. Table 3 shows the definition of the metrics mentioned.

Accuracy: *m* is the total number of samples. We use *c_i_* to represent the number of the *i*-th cluster points classified correctly. We predict the clusters *r* by performing clustering methods and then measure the accuracy according to the true label.

Adjusted Rand Index: [y_1_, y_2_, …, y_s_] stands for the true labels of datasets, while [c_1_, c_2_, …, c_r_] stands for the clusters separated by MDSVC. The sum of TP and TN that we need to obtain can represent the consistency between the clustering result and the result of the original cluster labels. We can distinctly compute it through the confusion matrix. The Rand index (RI), which equals (TP + TN)$/C_{2}^{m}$, represents the frequency of occurrence of agreements over all of the instance pairs. Finally, we can calculate the RI value. However, the RI value is not a constant close to zero for two random label assignments. The ARI, discounting the expected RI of random partition, can however address this issue.

### 4.2. Experimental Results and Analysis

In the process of SVC tuning, it is noted that there are often too many SVs or too few SVs, failing to form a better contour. Irrational SVs may not divide the clusters better and/or obtain higher precision. Based on this observation, we design experiments on the number of SVs with varying values of *λ*_1_ and *q*. As mentioned before, the Gaussian kernel *k*(*x*, *y*) = exp(−*q* *‖*x* − *y*‖^2^) is employed for nonlinear clustering, and we can derive *k*(*x*, *x*) = 1. We apply the commonly used dichotomy method to select the kernel width coefficient *q*.

**Figure 2:**
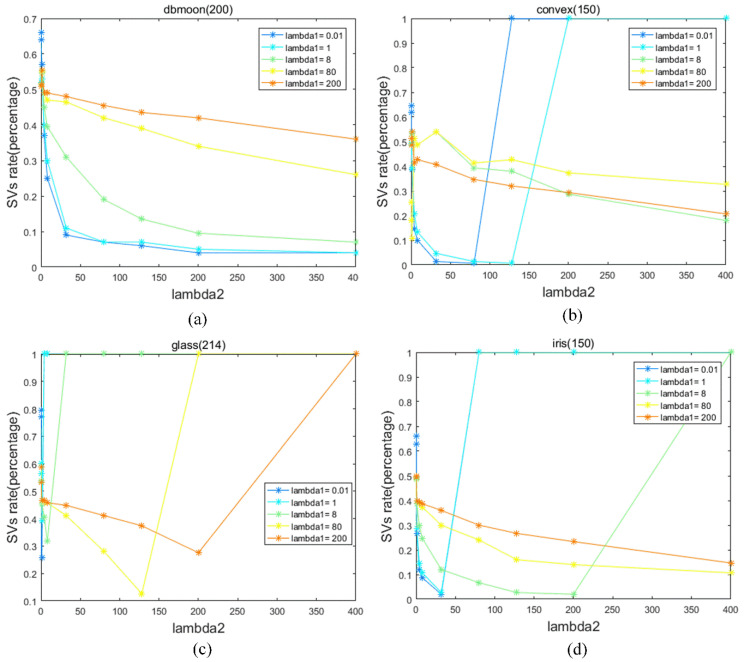
(**a**): The relationship between *λ*_1_ and *λ*_2_ on dbmoon about SVs. (**b**): The relationship between *λ*_1_ and *λ*_2_ on convex. (**c**): The relationship between *λ*_1_ and *λ*_2_ on glass about SVs. (**d**): The relationship between *λ*_1_ and *λ*_2_ on iris about SVs.

Before conducting experiments on the evaluations for MDVSC and other clustering methods, we analyze the relationship between *λ*_1_ and *λ*_2_ about SVs on two artificial datasets and two real datasets in Figure 2. For the appropriate range of these two parameters, we can realize that the number of SVs increases when *λ*_1_ increases as ***a*** is closer to the denser data in the feature space. Furthermore, the increase in *λ*_2_ leads to a decrease in the number of SVs for less volatility in terms of distance from ***a*** because the sphere is in the right place with fewer SVs. Thus, it is instructive for us to adjust *λ*_1_ and *λ*_2_ to solve the problem of too many or too few SVs when *q* and *C* are given.

We show the results with respect to the corresponding performance metrics in Table 4 and Table 5, where PERCENTAGE represents the percentage of the average number of SVs to the total data. We adopt/to represent the method has no need to compute the PERCENTAGE. We summarize the win/tie/loss counts for MDSVC in the last row compared with other methods. For a clearer comparison between MDSVC and SVC, *q* is selected from the same range [2^−7^, 2^7^] to compute the PERCENTAGE.

**Figure 3:**
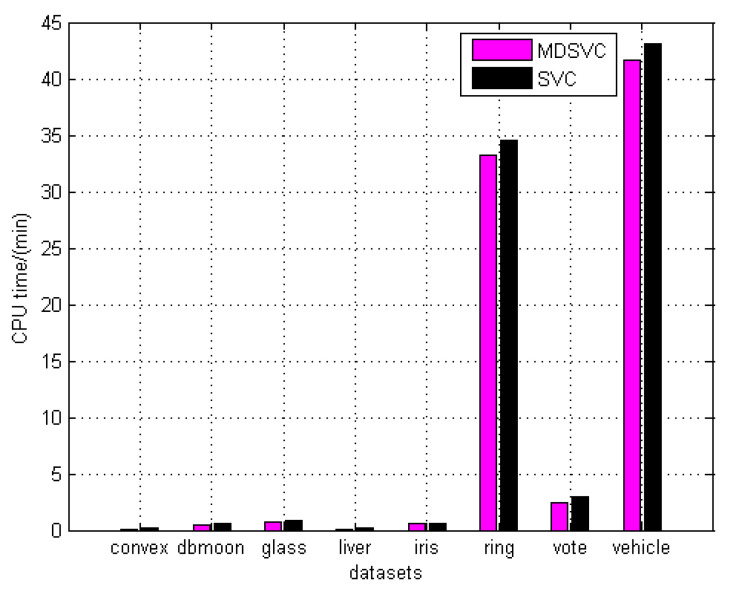
The CPU time of MDSVC and SVC.

In particular, the evaluation of datasets is shown in Table 4 and Table 5. Table 4 shows that MDSVC is almost on par with SVC on artificial datasets. It is worth noting that our MDSVC can reduce the number of SVs significantly under the same conditions compared to SVC, i.e., the same *q* and *C*. In Table 5, although we note that both SVC and MDSVC have worse Acc or ARI on some datasets, MDSVC still obtains better results than SVC and other methods on most real datasets. Based on the analysis of the experiments, we derive that we can change the SVs by changing the other parameters, *λ*_1_ and *λ*_2_, to achieve better performance when the parameters *q* and *C* are selected for MDSVC. In addition, in terms of the CPU time, MDSVC has superior performance on the datasets (ring, vehicle) with higher dimensions and larger size than SVC, as shown in Figure 3. Referring to the comparison of the CPU time between MDSVC and SVC, we indicate that MDSVC has two advantages: better performance and less running time.

**Figure 4:**
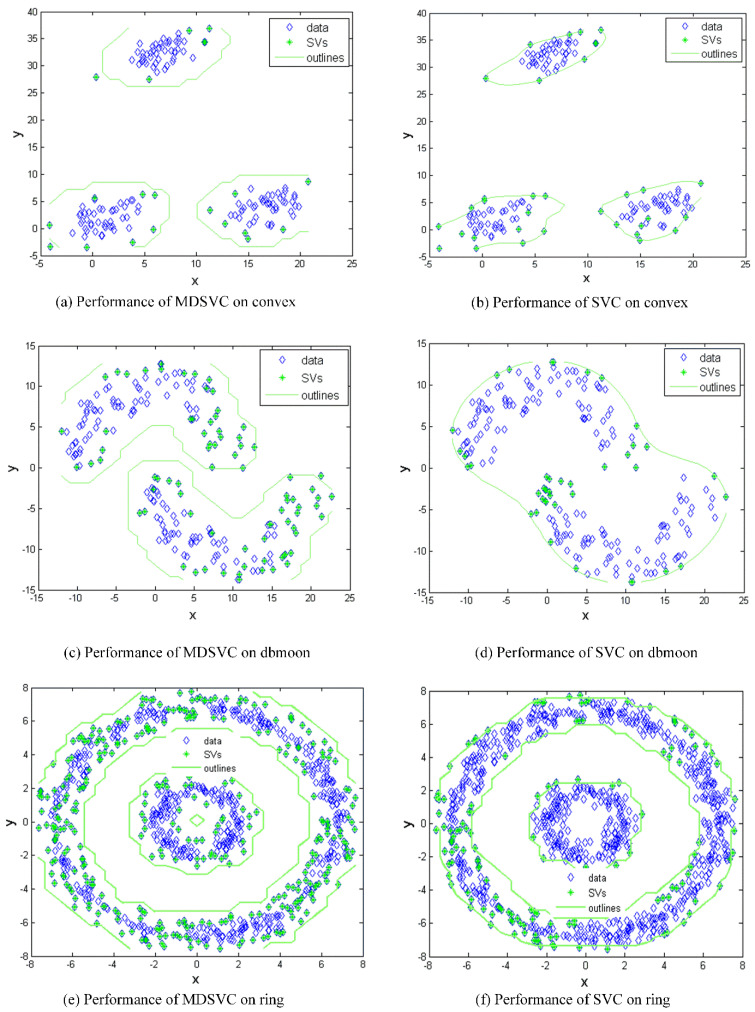
The result of MDSVC on three artificial datasets: convex, dbmoon, and ring. The parameters are set as follows: (**a**): *q* = 0.1; *λ*_1_= 8; *λ*_2_ = 32; *C* = 0.1. (**b**) *q* = 1; *C* = 0.1. (**c**): *q* = 0.1; *λ*_1_ = 1; *λ*_2_ = 4; *C* = 0.1. (**d**) *q* = 0.5; *C* = 0.1. (**e**): *q* = 2; *λ*_1_ = 200; *λ*_2_ = 300; *C* = 0.1. (**f**) *q* = 1; *C* = 0.5.

The estimated clustering assignments on artificial datasets, convex, and ring, are shown in Figure 4. In order to show the clusters divided by SVs more intuitively and accurately, we draw the contour lines decided by *R*. We note that the SVC algorithm is almost always overfitting on artificial datasets when the boundary is optimal; that is, all data points are identified as SVs, and, thus, Figure 4 only shows the best non-fitting effect of SVC. Obviously, MDSVC is superior to SVC in terms of forming better boundaries on artificial datasets.

Considering Figure 4a–d, the boundaries of the convex and the dbmoon formed by MDSVC are more rational than SVC in terms of separating clusters. For the ring set, the challenge for SVC is to make rational boundaries with the appropriate number of SVs. MDSVC forms four more rational boundaries and, thus, separates the ring set into two clusters, as shown in Figure 4e, while SVC recognizes only two boundaries in Figure 4f. Moreover, the introduction of statistical items (non-negative), which makes the hyperplane closer to the denser part in the feature space, results in the value of *R* being larger than SVC. Therefore, it can be seen that we have obtained a greater boundary under the premise of not increasing outliers. In summary, MDSVC obtains better boundaries and a better presentation of the statistical information in the above datasets.

**Figure 5:**
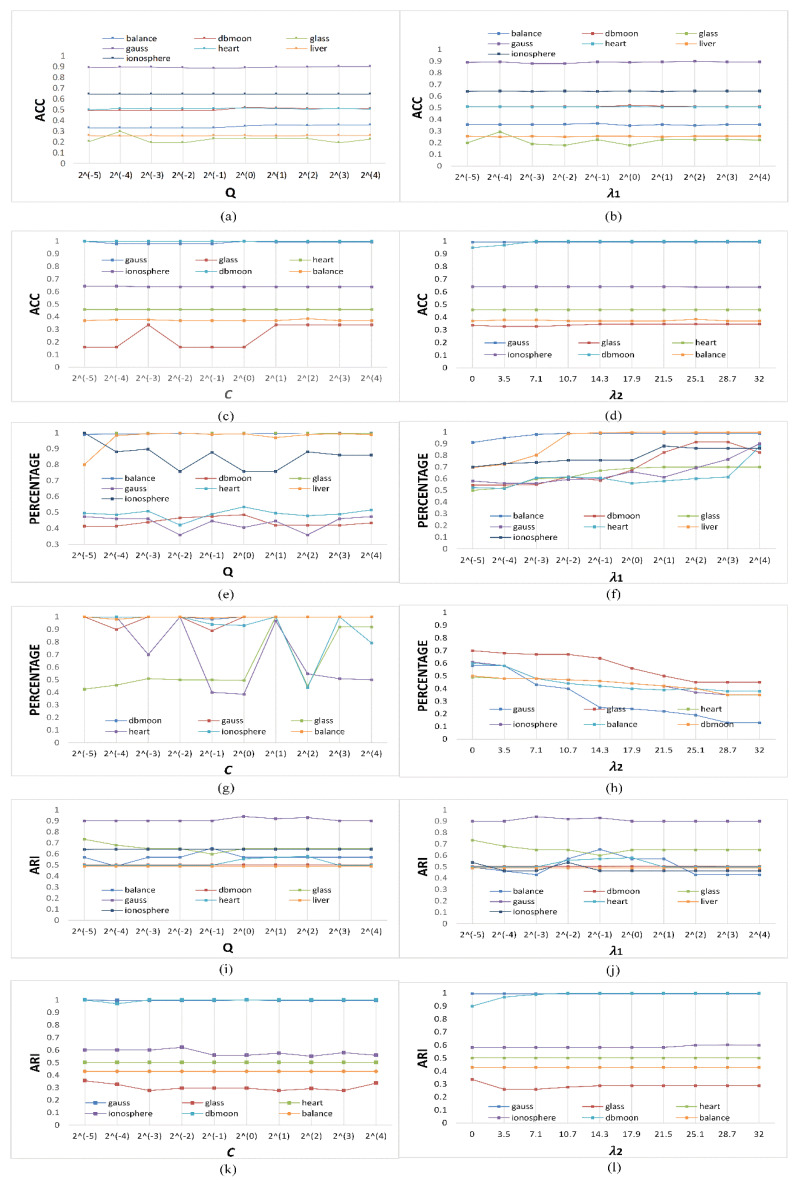
The impact of *λ*_1_, *λ*_2_, *C*, and kernel parameter Q on ARI, Acc, and PERCENTAGE for different datasets. (**a**–**d**): The impact of *λ*_1_, *λ*_2_, *C*, and kernel parameter Q on Acc. (**e**–**h**): The impact of *λ*_1_, *λ*_2_, *C*, and kernel parameter Q on PERCENTAGE. (**i**–**l**): The impact of *λ*_1_, *λ*_2_, *C*, and kernel parameter Q on Acc.

For further evaluation, we assess the impact of parameters on ARI, Acc, and PERCENTAGE as the change of parameter values may have a significant influence on the clustering results. Percentage characterizes the level of SVs. For our MDSVC, there are three trade-off parameters *λ*_1_, *λ*_2_, *C*, and the kernel parameter *q*. We show the impact of *λ*_1_ on ARI, Acc, and PERCENTAGE by varying it from 2^−5^ to 2^5^ while making the other parameters fixed as the optimal ones. As one can see from Figure 5e–h, the number of SVs is more sensitive to the kernel *q* and *C* compared to *λ*_1_ and *λ*_2_. In Figure 5b,d,f,h,j,l, we can see that the results are not sensitive to parameter *λ*_1_ after reaching the optimal results on most datasets. To sum up, we indicate that the mean and variance are both the main factors that affect the performance of the algorithm.

## 5. Discussion

It has been proved that trade-off parameters, *q* and *C*, have a significant impact on the results of SVC [5,7]. Obviously, we may spend more time in finding the optimal parameters that characterize a better boundary of clusters for SVC. This will result in a large number of SVs during the tuning process, which may affect the partition of clusters and is unreasonable, obviously. We know that it is feasible to adjust parameter *C* to obtain better performance, but it comes at the cost of increasing outliers. To solve these problems and inspired by the margin theory, we reconstruct a new hypersphere to identify the clusters to make denser sets more easily divided by employing the margin distribution, and then we establish the corresponding theory. We circumvent the high complexity resulting from the variance by demonstrating Theorem 1 and employing the Gaussian kernel, and then we derive the convex optimization problem.

As for the MDSVC algorithm, we design the customized DCD method to solve the convex optimization problem [25]. MDSVC has two other trade-off parameters compared to SVC, namely, *λ*_1_, *λ*_2_. Furthermore, we demonstrate that both of them play an important role in MDSVC through experiments shown in Figure 2 and equations about hypersphere we derive in Section 2. In Figure 4, we can obtain some useful instructive insights as an avenue for adjusting the number of SVs. Therefore, we can obtain better performance by increasing the *λ*_1_ value while there are few SVs. Moreover, we can increase *λ*_2_ value to reduce SVs. If one focuses on forming better outlines of clusters, the recommendation is to control the ratio of *λ*_1_ and *λ*_2_ to between 10^−2^ and 10^2^. Once the number of SVs changes drastically, there is no need for us to increase the value of *λ*_1_ and *λ*_2_. Meanwhile, what we should be aware of is that *λ*_1_ should not be zero. We further theoretically prove that the error has an upper bound in Section 3. Due to the lack of prior knowledge (true labels) of clustering algorithms, it is difficult for us to achieve our error bound in a manner similar to the approach used in LDM. We make it by taking the advantage of the error proposed in SVDD [6] and the lemma derived in CCL [9]. According to Figure 1b,c and Figure 4c–e, minimizing the mean and variance can make datasets properly outlined with a proper amount of SVs from a practical and theoretical perspective, while the outlines of SVC are inappropriate. However, we found that our method performed generally when the edge points of datasets are separated relatively densely, where edge points are a collection of relatively sparsely distributed points in the data space. Based on the experiments and formulas obtained; thus, we think that our method performs better on the datasets with edge points dispersing sparsely.

In short, the novel contribution of our work is that we redefine the hyperplane and the center in feature space considering the distribution of data to form better boundaries with a proper amount of SVs. Furthermore, experimental results in most datasets indicate that MDSVC achieves better performance, which further demonstrates the superiority of our method. In the future, we will design a corresponding method to improve the performance, which redefines the clustering partition.

## 6. Conclusions

In this research, we propose MDSVC, which employs the mean and variance, leveraged by marginal theory and SVM. The novelty of MDSVC lies in its reconstruction of the hyperplane, reducing the number of support vector points compared to SVC under the same conditions, and the improvement in generalization performance. We also have theoretically proven that our generalization performance has been improved, and the error has an upper bound. To optimize the objective function of MDSVC, we employ the DCD method with high applicability and efficiency. Experimental results in most datasets show that MDSVC achieves better performance, which indicates its superiority.

In our future work, we will study the partition of the second stage to further improve the performance of our method. At the same time, to assess the application potential of our algorithm, we will employ our model in more application scenarios.

## Figures and Tables

**Table 1:** Time Complexity Calculation of formulas involved.

The Formula of MDSVC	Time Complexity of the Formula
$Q=x^{T}x$	m*n*m
$H=\frac{8\lambda_{2}}{m}\sum_{i=1}^{m}Q_{i}Q_{i}{}^{T}$	m^3^
$P=\frac{8\lambda_{2}}{m^{2}}\sum_{i=1}^{m}\sum_{j=1}^{m}Q_{i}Q_{j}{}^{T}$	m^3^
$G={\left((\lambda_{1}+1)Q+H+P\right)}^{-1}Q$	m^3^
$A=\frac{\lambda_{1}}{m}{\left((\lambda_{1}+1)Q+H+P\right)}^{-1}Qe=\frac{\lambda_{1}}{m}Ge$	m^2^

**Table 2:** Experimental Datasets.

Source	Datasets	Samples	Feature
artificial	convex	150	3
	dbmoon	200	2
	ring	900	2
real	iris	150	3
	glass	214	9
	breast	277	9
	heart	303	13
	liver	345	6
	ionosphere	351	34
	vote	435	16
	balance	625	4

**Table 3:** Formula of metrics.

Metrics	Definition
Acc	$\mathrm{Acc}=\frac{\sum_{i=1}^{r}c_{i}}{m}$
ARI	$\mathrm{ARI}=\frac{\mathrm{RI}-E[\mathrm{RI}]}{\max(\mathrm{RI})-E[\mathrm{RI}]}\;$

**Table 4:** The result comparisons on artificial datasets.

Datasets	Metric	KM	SC	HC	ODMC	SVC	MDSVC
*convex*	ARI Acc PERCENTAGE	0.970 0.820 /	0.748 0.013 /	1.000 0.333 /	0.329 0.333 /	1.000 1.000 64.2%	1.000 1.000 35.0%
*dbmoon*	ARI Acc PERCENTAGE	0.638 0.900 /	0.324 0.185 /	0.516 0.140 /	0.498 0.500 /	0.928 0.990 79.7%	1.000 1.000 55.3%
*ring*	ARI Acc PERCENTAGE	0.113 0.322 /	0.171 0.338 /	1.000 0.500 /	0.420 0.511 /	1.000 1.000 95.8%	1.000 1.000 53.1%
MDSVC: w/t/l	ARI Acc PERCENTAGE	(3/0/0) (3/0/0) /	(3,0,0) (3,0,0) /	(3,0,0) (3,0,0) /	(3,0,0) (3,0,0) /	(1,2,0) (1,2,0) (3,0,0)	

**Table 5:** The result comparisons on real datasets.

Datasets	Metric	KM	SC	HC	ODMC	SVC	MDSVC
*iris*	ARI	0.730	0.474	0.558	0.329	0.848	0.828
	Acc	0.347	0.193	0.333	0.333	0.667	0.753
	PERCENTAGE	/	/	/	/	96.1%	51.8%
*glass*	ARI	0.230	0.067	0.259	0.260	0.750	0.751
	Acc	0.327	0.014	0.028	0.327	0.289	0.351
	PERCENTAGE	/	/	/	/	89.8%	12.5%
*breast*	ARI	0.171	0.177	0.062	0.585	0.542	0.612
	Acc	0.376	0.087	0.025	0.707	0.484	0.711
	PERCENTAGE	/	/	/	/	98.7%	71.5%
*heart*	ARI	0.564	0.074	0.058	0.637	0.571	0.580
	Acc	0.551	0.172	0.195	0.772	0.990	0.990
	PERCENTAGE	/	/	/	/	61.3%	55.1%
*liver*	ARI	0.001	0.002	0.009	0.511	0.489	0.512
	Acc	0.154	0.033	0.067	0.420	0.476	0.493
	PERCENTAGE	/	/	/	/	89.7%	50.4%
*ionosphere*	ARI	0.178	0.191	0.189	0.612	0.747	0.756
	Acc	0.477	0.393	0.171	0.738	0.687	0.734
	PERCENTAGE	/	/	/	/	90.6%	26.2%
*vote*	ARI	0.296	0.009	0.512	0.525	0.512	0.525
	Acc	0.540	0.112	0.356	0.386	0.361	0.387
	PERCENTAGE	/	/	/	/	95.2%	88.7%
*balance*	ARI	0.114	0.184	0.695	0.112	0.570	0.653
	Acc	0.294	0.075	0.016	0.147	0.278	0.356
	PERCENTAGE	/	/	/	/	61.4%	58.1%
MDSVC: w/t/l	ARI	(7,0,0)	(7,0,0)	(6,0,1)	(4,2,1)	(5,1.1)	
	Acc	(6,0,1)	(7,0,0)	(7,0,0)	(5,1,1)	(6,1,0)	
	PERCENTAGE	/	/	/	/	(7,0,0)	

## Data Availability

The implementation is publicly available at http://github.com/Galichen/MDSVC (accessed on 3 November 2021).

## Appendix A. Support Vector Clustering

Support vector clustering (SVC) introduces soft boundary as a tolerance mechanism to reduce the number of boundary support vector points. The algorithm is robust to noise and does not need to know the number of clusters. However, the effectiveness of the algorithm depends on the selection of the kernel width coefficient *q* and the soft boundary constant *C*. Clearly, parameter adjustment is time-consuming. SVC has the formulation as follows

(A1) $$\begin{array}{l} \underset{R,a}{\min}R^{2}+C\sum_{i=1}^{m}\xi_{i}\\ \;\;s.t.{\left\|\phi (x_{i})-a\right\|}^{2}\leq R^{2}+\xi_{i},\xi_{i}\geq 0 \end{array}$$

where parameter *C* is used for controlling outliers and $C\sum_{i=1}^{m}\xi_{i}$ is a penalty term, and then the slack variables $\xi_{i}$ are used as tolerance. SVC looks for the smallest enclosing sphere of radius *R*, under the constraints ${\left\|\phi (x_{i})-a\right\|}^{2}\leq R^{2}+\xi_{i}$, where ||.|| is the Euclidean norm and ***a*** is the center of the hypersphere. We can use the Lagrange function to solve the problem

$$L=R^{2}+C\sum_{i=1}^{m}\xi_{i}-\sum_{i=1}^{m}\mu_{i}\xi_{i}-\sum_{i=1}^{m}\beta_{i}(R^{2}+\xi_{i}-{\left\|\phi (x_{i})-a\right\|}^{2})$$

After we take the derivative of the above formula, the dual problem can be cast as follows

$$\begin{array}{l} \underset{\beta }{\max}L=\sum_{i}\beta_{i}\kappa (x_{i},x_{i})-\sum_{i}\sum_{j}\beta_{i}\beta_{j}\kappa (x_{i},x_{j})\\ 0\leq \beta_{i}\leq C \end{array}$$

Thus, we can define the distance of each point in the feature space

$$R^{2}(x)={\left\|\phi (x)-a\right\|}^{2}$$

Finally, *R*^2^ has the following form

(A2) $$R^{2}(x)=\kappa (x,x)-2\sum_{i}\beta_{i}\kappa (x,x_{i})+\sum_{i,j}\beta_{i}\beta_{j}\kappa (x_{i},x_{j})$$

The radius of the hypersphere is

$$R=\left\{R(x_{i})|x_{i}\;\mathrm{is}\;a\;\mathrm{support}\;\mathrm{vector}\right\}$$

Here, the Lagrange multiplier $\beta_{i}\in (0,C)$, ***x_i_*** is a support vector (SV). The point is a boundary support vector point (BSV) when $\beta_{i}=C$. SVC used the adjacency matrix ***A_ij_*** to identify the connected components. For two points ***x_i_*** and ***x_j_***

$$A_{ij}=\left\{ \begin{array}{l} 0\;\;\exists x,s.t.\;\;\;\;R^{2}\left(x\right)>R,\mathrm{and}\;x-x_{i}=t\left(x_{j}-x_{i}\right)\\ 1\;\;\mathrm{otherwise}. \end{array}\right.$$

Finally, the clusters can be defined according to the adjacency matrix ***A_ij_*_._** The time complexity of calculating the adjacency matrix is *O* (*vm^2^*), in which v is the number of samples for the line segment. The quadratic programming problem can be solved by the SMO algorithm, the memory requirements of which are low, and it can be implemented using *O* (1) memory at the cost of a decrease in efficiency. The obvious shortcoming of SVC lies in the high cost of partition.
